# Cost-effectiveness of maintenance niraparib with an individualized starting dosage in patients with platinum-sensitive recurrent ovarian cancer in China

**DOI:** 10.3389/fphar.2023.1198585

**Published:** 2023-07-27

**Authors:** Yin Shi, Di Xiao, Shuishi Li, Shao Liu, Yu Zhang

**Affiliations:** ^1^ Department of Pharmacy, Xiangya Hospital, Central South University, Changsha, China; ^2^ National Clinical Research Center for Geriatric Disorders, Xiangya Hospital, Central South University, Changsha, China; ^3^ The Hunan Institute of Pharmacy Practice and Clinical Research, Changsha, China; ^4^ Department of General Surgery, Xiangya Hospital, Central South University, Changsha, China; ^5^ Department of Gynecology, Xiangya Hospital, Central South University, Changsha, China; ^6^ Gynecological Oncology Research and Engineering Center of Hunan Province, Changsha, China

**Keywords:** niraparib, routine surveillance, ovarian cancer, maintenance therapy, cost-effectiveness

## Abstract

**Objective:** Niraparib improved survival in platinum-sensitive recurrent ovarian cancer (PSROC) patients *versus* routine surveillance, accompanied by increased costs. Based on the NORA trial, we evaluated for the first time the cost-effectiveness of maintenance niraparib with individualized starting dosage (ISD) in China.

**Methods:** A Markov model was developed to simulate the costs and health outcomes of each strategy. The total costs, quality-adjusted life years (QALYs), and incremental cost-effectiveness ratios (ICERs) were measured. One-way and probabilistic sensitivity analysis were performed to estimate model robustness. Scenario analyses were also conducted.

**Results:** Compared to routine surveillance, niraparib additionally increased QALYs by 0.59 and 0.30 in populations with and without germline *BRCA* (g*BRCA*) mutations, with incremental costs of $10,860.79 and $12,098.54, respectively. The ICERs of niraparib over routine surveillance were $18,653.67/QALY and $39,212.99/QALY. At a willingness-to-pay (WTP) threshold of $37,488/QALY, the ISD enhanced the likelihood of cost-effectiveness from 9.35% to 30.73% in the g*BRCA*-mutated group and from 0.77% to 11.74% in the non-g*BRCA* mutated population. The probability of niraparib being cost-effective in the region with the highest *per capita* Gross Domestic Product (GDP) in China was 74.23% and 76.10% in the g*BRCA*-mutated and non-g*BRCA* mutated population, respectively. Niraparib was 100% cost-effective for National Basic Medical Insurance beneficiaries under the above WTP thresholds.

**Conclusion:** Compared to routine surveillance, the ISD of niraparib for maintenance treatment of PSROC is cost-effective in the g*BRCA*-mutated population and more effective but costly in the non-g*BRCA* mutated patients. The optimized niraparib price, economic status, and health insurance coverage may benefit the economic outcome.

## 1 Introduction

Ovarian cancer ranks eighth in incidence and mortality among female malignancies (Sung et al., 2021). According to the 2020 GLOBOCAN Global Cancer Data, there were 313,959 newly diagnosed ovarian cancer cases and 207,252 deaths worldwide. In China, ovarian cancer is the third most prevalent malignancy of the female reproductive system and a serious threat to women’s health (Zheng et al., 2022).

Ovarian cancer is characterized by an insidious onset, a high recurrence rate, a short patient survival period, and progressive resistance to multiline chemotherapy (Hanker et al., 2012; Zhang et al., 2022). Several factors, including stage, histological type, molecular characteristics, as well as treatment strategies, influence the prognosis of ovarian cancer (Liu et al., 2021). Approximately 90 percent of primary ovarian malignancies are epithelial, with high-grade serous carcinoma (HGSC) being the predominant histological type of epithelial ovarian cancer (EOC) (Cheung et al., 2022). HGSC is categorized as type II EOC that exhibits more aggressive, and harbors a defect in at least one DNA damage response (DDR) pathway (Pavlidis et al., 2021; Ovejero-Sanchez et al., 2023). It was not until the discovery of poly-ADP-ribose polymerase (PARP) inhibitors that the pattern of EOC treatment could be altered. PAPR inhibitors induce tumor cell death through a “synthetic lethal” mechanism, which is particularly effective in individuals with *BRCA*1 and/or 2 mutations or other homologous recombination deficiency (HRD) (Lau et al., 2022). It was identified that 23.8% of unselected Chinese patients with EOC carried BRCA1/2 mutations (20.3% germline and 4.1% systemic) (You et al., 2020). In addition, the synthetic lethal interaction may be exploited outside of germline BRCA mutations in the context of HRD, and research in this area is ongoing. In the absence of homologous recombination repair function, DNA double-strand breaks will be processed by alternative but error-prone repair pathways, such as non-homologous end joining repair (NHEJ), resulting in genomic instability and ultimately the death of cancer cells (Boussios et al., 2022).

PARP inhibitor maintenance therapy after initial chemotherapy or platinum-sensitive relapse therapy has been shown to enhance progression-free survival (PFS) to varying degrees, thus promoting prolonged survival in some patients (Tattersall et al., 2022). The Chinese National Medical Products Administration has approved olaparib, niraparib, and fluzoparib as maintenance treatments for recurrent ovarian cancer. Among them, niraparib is the only one with biomarker-independent and all-comer benefits. The first phase III trial of niraparib in China, namely, the NORA trial (NCT03705156), utilized an individualized starting dosage (ISD) of niraparib for platinum-sensitive recurrent ovarian cancer (PSROC) maintenance (Wu et al., 2021). Compared to placebo, maintenance niraparib prolonged the median PFS in patients with germline *BRCA* (g*BRCA*) mutations (5.5 months vs not reached) and without g*BRCA* mutations (3.9 vs 11.1 months) (Wu et al., 2021). Recently, *ad hoc* interim overall survival (OS) results demonstrated a certain degree of OS benefit in a g*BRCA*-mutated population (47.61 months vs not reached) and a non-g*BRCA* mutated group (38.41 vs 43.10 months) (Mirza et al., 2023). However, extended survival is accompanied by high drug costs and the expense associated with adverse event (AE) management.

Despite its significant clinical benefits, the economic burden of maintenance niraparib on both patients and society is a prominent concern. In this study, we examined for the first time the cost-effectiveness of maintenance niraparib ISD *versus* routine surveillance in PSROC patients classified by g*BRCA* status from the perspective of the Chinese healthcare system.

## 2 Methods

### 2.1 Model overview

Our model simulation study used data from a published trial. No human participants were involved in this study, and no institutional review board approval by an ethics committee was needed. Economic evaluations were based on the Consolidated Health Economic Evaluation Reporting Standards (CHEERS) (Husereau et al., 2022).

**FIGURE 1:**
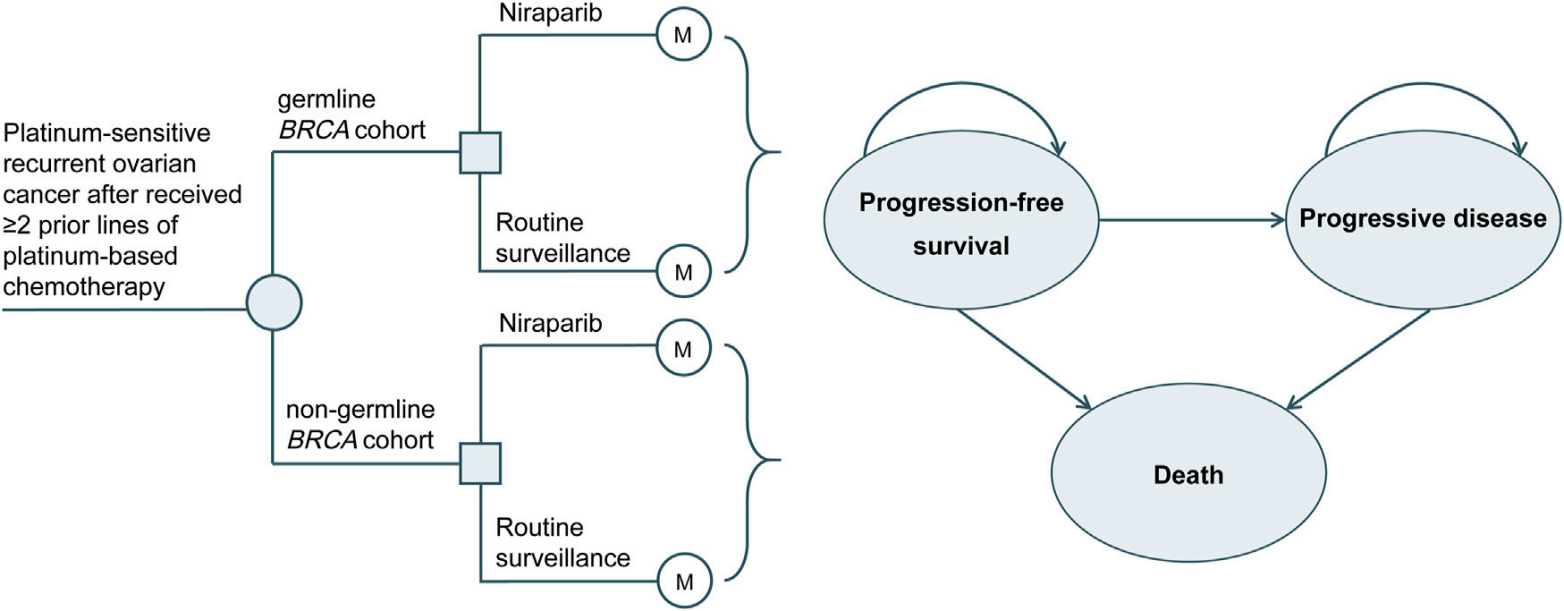
Markov model.

In this study, TreeAge Pro 2021 (TreeAge Software, Williamstown, MA, United States) was applied to develop a 3-state Markov model to simulate the costs and health outcomes of maintenance niraparib or routine surveillance for PSROC (Figure 1). The primary outcomes included life years (LYs), quality-adjusted life years (QALYs), total costs, and incremental cost-effectiveness ratios (ICERs). Based on the World Health Organization (WHO) recommendations, the willingness-to-pay (WTP) threshold in this study was equal to 3 times China’s gross domestic product (GDP) *per capita* in 2021 ($37,488) (World Health Organization, 2021).

The cycle length was 4 weeks, consistent with the dosing cycle in the NORA trial. The time horizon was 21.3 years, during which more than 99.99% of patients died in both arms. The characteristics of the simulated population were consistent with those of the NORA trial population (Wu et al., 2021). Patients received either maintenance treatment with niraparib (300 mg orally once daily for those with a body weight ≥77 kg and platelet count ≥150×10^3^/µL, otherwise 200 mg orally once daily) or routine surveillance until disease progression or intolerable toxicity. Upon progression, patients in both arms were permitted to receive subsequent treatment until death. Due to the unavailability of subsequent therapy in both arms of the NORA trial, the choice of follow-up treatment was assumed from the published literature (Poveda et al., 2021) combined with clinical experience (Supplementary Table S1). Intervention discontinuation associated with AEs was 4% and 5.70% in the niraparib and routine surveillance arms, respectively (Wu et al., 2021). We applied a half-cycle correction, and a discount rate of 5% per year to the cost and effect outcomes (Chinese Pharmaceutical Association, 2020).

### 2.2 Model probabilities

All patients entered the research in the PFS state, and the subsequently observed states were PFS, progressing disease (PD), or death. The probabilities of PD and death from platinum-sensitive recurrent state for the niraparib and routine surveillance arms in the g*BRCA*-mutated and non-g*BRCA* mutated populations were calculated based on the Kaplan-Meier PFS and OS curves from the NORA study (Wu et al., 2021; Mirza et al., 2023). The individual patient data were recreated using the method of Hoyle and Henley (2011). We implemented GetData Graph Digitizer software to extract data points for the PFS and OS curves, and then the recreated PFS/OS curves were fitted to the following parametric survival functions: exponential, Weibull, log-logistic, lognormal, generalized gamma, gamma, and Gompertz distributions. The best-fit distribution for each curve was chosen according to the lowest value of the Akaike information criterion and the Bayesian information criterion, combined with a visual inspection. Details of the survival models of niraparib and routine surveillance in the g*BRCA*-mutated and non-g*BRCA* mutated cohorts with PSROC are shown in Supplementary Table S2, Supplementary Figure S1, Supplementary Figure S2.

### 2.3 Cost and health state utility values

Only direct medical costs, such as drug costs, intravenous chemotherapy, serious adverse event (SAE) management, g*BRCA* mutation tests, follow-up, and terminal care, were examined in this study (Supplementary Table S1). Drug prices were collected from the payment standards in China’s 2021 national insurance drug list and the winning bid prices in the drug procurement system (The State Council the People’s Republic of China, 2021; Pharmaceutical Classified Procurement System of Hunan Province, 2022). The expenses of SAE management and follow-up were estimated from the clinical experience. The costs of chemotherapy intravenous infusion and g*BRCA* mutation testing were based on current local pricing, while the remaining costs were sourced from prior cost-effectiveness studies (Li et al., 2020). Using China’s consumer price index, we adjusted all costs to their equal value in U.S. dollars for the year 2021 (1 US dollar equates to 6.45 Chinese yuan) (National Bureau of Statistics of China, 2022).

For administration dosage, we used a standard AUC of 5 mg/mL/min, with the assumption that an average female weighs 61 kg and has a body surface area of 1.64 m^2^ (Wu et al., 2021). SAEs occurring in greater than 10% of patients in either strategy and with an occurrence difference of more than 5% between the groups were assessed in this study, including anemia, thrombocytopenia, and neutropenia. It was supposed that all SAEs were experienced in the first cycle of the model.

For the niraparib and routine surveillance groups, the PFS state utility values were 0.849 and 0.820, respectively, and the PD state utility values were 0.793 and 0.775, respectively, based on literature assumptions (Guy et al., 2019). According to the published literature, neutropenia, anemia, and thrombocytopenia do not cause a significant decrease in quality of life, so disutility values for AEs were not considered in this study (Oza et al., 2018).

### 2.4 Sensitivity and scenario analyses

We explore the influence of model parameters on the robustness of the results through sensitivity analysis, including one-way sensitivity analysis and probabilistic sensitivity analysis. The parameter range in the one-way sensitivity analysis was determined by either the reported 95% confidence interval or a ±20% change from the baseline value, except for the drug price parameter, for which the range was based on market fluctuations (Supplementary Table S1).

Ten thousand Monte Carlo simulations were used to perform probabilistic sensitivity analyses, in which critical model parameters were simultaneously varied in a specific type of distribution. Cost parameters were assumed to obey a gamma distribution, and probability parameters and utility values were assumed to obey a beta distribution.

Furthermore, we conducted scenario analyses to investigate the effect of fixed dosing, economic development level, and enrollment in the National Basic Medical Insurance program on the cost-effectiveness of niraparib. Scenario 1: We assumed that all patients received a fixed starting dosage (FSD) of 300 mg/day regardless of body weight and platelet count. Scenario 2: Due to the large wealth gap between regions in China, we added a scenario analysis at WTP thresholds of $19,002/QALY (Gansu, the province with the lowest GDP in China) and $85,176/QALY (Beijing, the city with the highest GDP in China). Scenario 3: Considering that most cancer treatment drugs are included in China’s 2021 national insurance drug list released by the National Healthcare Security Administration (The State Council the People’s Republic of China, 2021), we included the out-of-pocket drug prices after a certain reimbursement percentage.

## 3 Results

### 3.1 Base-case analysis

For the g*BRCA*-mutated population, an additional 0.59 QALY (0.56LY) was obtained with maintenance niraparib compared to routine surveillance, accompanied by an incremental cost of $10,860.79 and an ICER of $18,653.67/QALY ($19,412.87/LY). For the non-g*BRCA* mutated cohort, the incremental cost of maintenance niraparib was $12,098.54 compared to routine surveillance, with an incremental effect of 0.30 QALY (0.26LY) and an ICER of $39,212.99/QALY ($46873.79/LY) (Table 1).

**TABLE 1:** Base case results.

Strategies	Cost, $	QALYs	LYs	ICER, $/QALY	ICER, $/LY
**g*BRCA*m**					
Niraparib	33,509.01	3.39	4.15	18,653.67	19,412.87
Routine surveillance	22,648.22	2.80	3.59		
**Non-g*BRCA*m**					
Niraparib	28,823.11	2.96	3.65	39,212.99	46,873.79
Routine surveillance	16,724.57	2.66	3.39		

Abbreviation: QALYs, quality-adjusted life years; LYs, Life years; ICERs, incremental cost-effectiveness ratios; g*BRCA*m, germline *BRCA* mutation.

### 3.2 Sensitivity analysis

**FIGURE 2:**
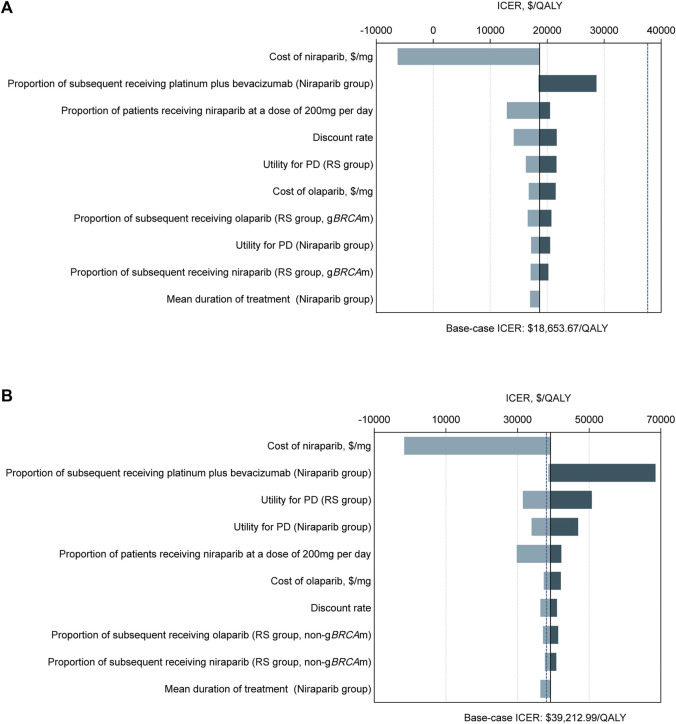
Tornado diagrams of one-way sensitivity analyses with greatest influence variables. The diagram shows the association of variables with the ICER of niraparib *versus* routine surveillance for platinum-sensitive recurrent ovarian cancer in **(A)** the g*BRCA*m cohort and **(B)** the non-g*BRCA*m cohort. The black vertical line represents the base-case result of $18,653.67 per QALY and $39,212.99 per QALY in the g*BRCA*m cohort and non-g*BRCA*m cohort, respectively. The blue vertical dotted line represents the WTP threshold of $37,488 per QALY. Abbreviation: ICER, incremental cost-effectiveness ratios; QALY, quality-adjusted life years; PD, progressed disease; RS, routine surveillance; g*BRCA*m, germline *BRCA* mutation.

The tornado diagram demonstrated the outcomes of the one-way sensitivity analysis (Figure 2). For the g*BRCA*-mutated population, the expense of niraparib had the dominant impact on the ICER, followed by the proportion of patients receiving niraparib at 200 mg per day and the discount rate. Regardless of the change in any parameter within the given range, the ICER was always under the WTP threshold ($37,488/QALY). In the non-g*BRCA* mutated cohort, the most influential modeling variable was also the niraparib cost, while the other sensitive parameters were the proportion of patients subsequently receiving platinum plus bevacizumab in the niraparib group, the utility for PD states in the routine surveillance group and the niraparib group, and the proportion of patients receiving niraparib at a daily dose of 200 mg. Maintenance therapy was found to be cost-effective when the price of niraparib was below $0.2320/mg.

**FIGURE 3:**
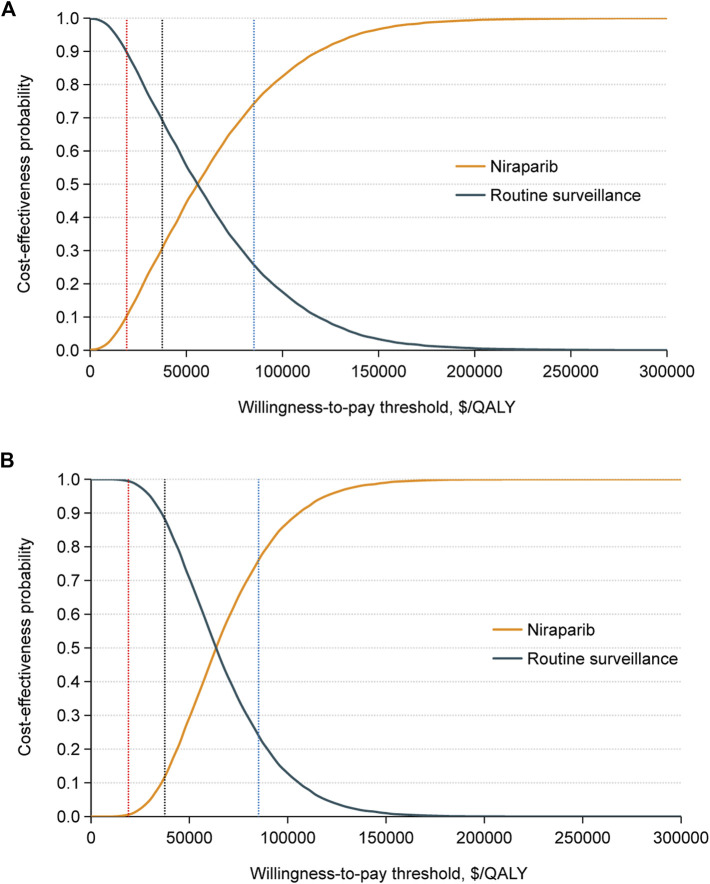
Cost-effectiveness acceptability curves for the niraparib and routine surveillance groups in **(A)** the g*BRCA*m cohort and **(B)** the non-g*BRCA*m cohort generated from the probabilistic sensitivity analysis (10,000 iterations). The red, black, and blue vertical dotted lines represent the $19,002, $37,488, and $85,176 per QALY. Abbreviation: QALY, quality-adjusted life years.

In the probabilistic sensitivity analysis, the acceptability curves indicated that in the g*BRCA*-mutated population, the possibility of having cost-effectiveness in the niraparib group was 30.73% at a WTP threshold of $37,488 per QALY, and the likelihood was over 50% that the niraparib group would be cost-effective at a WTP threshold greater than $55,820 per QALY. For the non-g*BRCA* mutated cohort, when the WTP threshold reached $37,488/QALY, the possibility of niraparib being cost-effective was 11.74%, and this value reached 50% at $63,622/QALY (Figure 3).

### 3.3 Scenario analyses

Scenario 1: When administered at an FSD of 300 mg/day, the ICER for niraparib *versus* placebo in the g*BRCA*-mutated population was $33,009.06/QALY. This was cost-effective at the current WTP ($37,488/QALY) but significantly more costly than individualized dosing ($19,218.97 vs $10,860.79) (Supplementary Table S3). Probabilistic sensitivity analysis showed an economic-benefit probability of 9.35%, which was much lower than the ISD probability of 30.73% (Supplementary Figure S3). The fixed dosage of niraparib was not cost-effective for the non-g*BRCA* mutated population (ICER of $62,763.81/QALY) when administered at 300 mg/day (Supplementary Table S2). The probability sensitivity analysis revealed that the likelihood of cost-effectiveness was only 0.77% (Supplementary Figure S3).

Scenario 2: When the WTP threshold was $19,002 per QALY, the possibility of maintenance niraparib proving cost-effective was 10.25% for the g*BRCA*-mutated population and 0.55% for the non-g*BRCA* mutated population. When the WTP threshold was set at $85,176 per QALY, the likelihood of maintenance niraparib having cost-effectiveness was 74.23% in the g*BRCA*-mutated population and 76.10% in the non-g*BRCA* mutated cohort (Figure 3).

Scenario 3: After medical insurance coverage, the out-of-pocket payments for niraparib, paclitaxel, carboplatin, bevacizumab, albumin-bound paclitaxel, olaparib, and letrozole would be discounted to 20%, 0%, 0%, 30%, 20%, 30%, and 5%, respectively. When considering the out-of-pocket price after medical insurance reimbursement, the ICER of niraparib compared to routine surveillance was $3,372.19/QALY in the g*BRCA*-mutated population and $5,643.96/QALY in the non-g*BRCA* mutated population (Supplementary Table S4). Probabilistic sensitivity analysis suggested that maintenance niraparib is totally cost-effective relative to routine monitoring, regardless of the g*BRCA* mutation status (Supplementary Figure S4).

## 4 Discussion

The promising results of the NORA study, which is the only second-line maintenance treatment based on ISD for Chinese PSROC patients, indicate considerable therapeutic benefits (Wu et al., 2021). Additionally, the high economic burden on patients and society is a growing issue for Chinese authorities.

This study demonstrated that compared to routine surveillance, maintenance niraparib was cost-effective for patients with g*BRCA* mutations. In contrast, niraparib was only cost-effective for the non-g*BRCA* mutated population if it cost less than $0.232 per milligram. Probabilistic sensitivity analysis revealed that at the current WTP threshold of $37,488/QALY, niraparib was cost-effective for 30.73% of the g*BRCA*-mutated population and 11.74% of the non-g*BRCA* mutated group.

Several model-based economic studies have investigated the cost-effectiveness of niraparib as second-line maintenance treatment for ovarian cancer. Four of them were based on the healthcare system (Zhong et al., 2018), society (Dottino et al., 2019), or payer (Guy et al., 2019) perspective in the United States, one on the single-payer perspective of Taiwan China (Leung et al., 2022), and one on the mainland Chinese healthcare system perspective (Nie et al., 2022). Due to differences in modeling approaches, population characteristics, drug pricing, WTP thresholds, national conditions and so on, the results of pharmacoeconomic studies in one country or region cannot be simply replicated and applied to others (Drummond et al., 2009). The study conducted in Taiwan, China, revealed that maintenance niraparib was cost effective in patients with g*BRCA* mutations but not in non-g*BRCA* mutated patients (Leung et al., 2022). The study by Nie J et al. included only a g*BRCA*-mutated population and showed that compared to routine surveillance, niraparib was cost-effective from the perspective of the Chinese healthcare system (Nie et al., 2022). The previous two studies, however, were based on clinical data from the NOVA trial using a fixed dosage of niraparib in a non-Chinese population, which could bias the results. Additionally, the model time horizon for these two studies was just 2 and 5 years, respectively.

To our knowledge, this is the first study to assess the cost-effectiveness of maintenance niraparib with an ISD for PSROC in the g*BRCA-*mutated and non-g*BRCA* mutated populations from the perspective of the mainland Chinese healthcare system. The population characteristics and clinical outcomes in this study were from the NORA trial, the only Chinese population-based randomized controlled trial of niraparib. This made it more applicable to the Chinese population and might decrease population bias. Meanwhile, we conducted a scenario analysis for the first time to investigate the effect of FSD, economic development level and enrollment in the National Basic Medical Insurance program on the cost-effectiveness of niraparib.

Compared to the FSD of niraparib, the ISD reduced the prevalence of adverse events and treatment discontinuation or interruption due to adverse reactions without compromising efficacy (Mirza et al., 2016). In addition, it lowered drug costs and AE management expenses, with drug costs having the greatest impact on the economic model in our study. This could explain the scenario analysis results that there was a lower probability of cost-effectiveness with fixed dosing of niraparib compared to individualized dosing, regardless of the *BRCA* mutation status.

As one of the world’s largest developing countries, China’s economic development varies by region. The highest *per capita* GDP in China was $28,392 in Beijing, while the lowest was $6,334 in Gansu Province. In this study, the WTP threshold was set at 3 times the national GDP *per capita*, as recommended by the WHO(World Health Organization, 2021). This may not accurately reflect the acceptability of each region nationwide. Thus, we included a WTP threshold analysis with a range from 3 times Gansu Province’s GDP *per capita* to 3 times Beijing’s ($19,002/QALY to $85,176/QALY). The results suggested that the probability of niraparib having an economic advantage may be greatly improved for patients in regions with higher levels of economic development in China, independent of *BRCA* status. In regions with lower levels of economic development, niraparib has a lower probability of being cost-effective in the *gBRCA*-mutated population and is almost not cost-effective in the non-*gBRCA* mutated population.

One effective way to improve cost-effectiveness may be to reduce the cost of antineoplastic agents by negotiating trade-offs in drug expenditure and coverage. Currently, niraparib is significantly less expensive in China than in developed countries such as the United States (Chan et al., 2022). This stems from our unique national health insurance negotiation system. In 2015, China introduced a pricing negotiation approach for patented and exclusive medicine involving pharmaceutical corporations and other stakeholders (The State Council the People’s Republic of China, 2015). The Interim Measures for the Administration of the National Basic Medical Insurance Drugs, applied in 2020, require an economic evaluation for drugs admitted to or removed from the medical insurance list as well as broadening of the spectrum of limited payment (The State Council the People’s Republic of China, 2020). In 2021, the National Health Security Administration negotiated with manufacturers to decrease the cost of niraparib by 81%, from $1.29/mg to $0.24/mg. This has drastically reduced the economic burden for Chinese patients with ovarian cancer. In addition, almost 95% of the Chinese population has access to the National Basic Medical Insurance, which covers approximately 1.4 billion people (National Healthcare Security Administration, 2021). Depending on the type of health insurance, the National Basic Medical Insurance program provides 20%–80% savings off the $0.24/mg cost of niraparib. Our study demonstrates that when only out-of-pocket prescription expenses are considered, the probability of niraparib being cost-effective increases to 100%. This will significantly expand the number of patients who benefit from maintenance niraparib.

Additionally, we must note the limitations of this study. First, as the final OS results were not yet available, we utilized the newly published *ad hoc* interim OS results from the NORA trial, and the maturity of OS curves was less than 50% (Mirza et al., 2023). The use of final OS results would be preferred because it would reduce the uncertainty of the model’s predicted outcomes. Second, since data on subsequent treatment after progression with niraparib were not yet available from the NORA trial, the subsequent treatment regimens and ratios in this paper were referenced from the published literature and the practical experience of clinical experts, which may cause bias. Third, based on the NORA trial, PARP inhibitors were used in 54% of the g*BRCA*-mutated population and 36% of the non-g*BRCA* mutated population in this study after disease progression in the routine surveillance group, which may lead to an overestimation of survival and effect of the routine surveillance group. In the real world, patients in the routine surveillance group frequently receive PARP inhibitors after disease progression. Fourth, Due to the lack of data, we explored the cost-effectiveness of FSD of niraparib in scenario 1 analysis using data from the NORA study, assuming the same baseline characteristics of niraparib FSD and ISD administration. Indeed, patients administered with FSD and ISD may differ in the incidence of adverse events, treatment interruption or discontinuation, and even prognosis, which may have biased the results. To verify model stability, we conducted one-way sensitivity analyses.

## 5 Conclusion

Our study demonstrated that compared with routine surveillance, maintenance niraparib with an ISD is cost-effective for patients with g*BRCA* mutations in China. For the non-g*BRCA* mutated population, niraparib is more effective but costly than routine surveillance, and the price reduction will benefit its cost-effectiveness. Economic outcomes could be further improved for patients receiving ISD, for those in regions with higher *per capita* GDP in China, or for those covered by the National Basic Medical Insurance program, independent of *BRCA* status.

## Data Availability

The original contributions presented in the study are included in the article/Supplementary Material, further inquiries can be directed to the corresponding authors.
