# Supplementary material for: Cost-effectiveness of maintenance niraparib with an individualized starting dosage in patients with platinum-sensitive recurrent ovarian cancer in China
Source: Front Pharmacol. 2023 Jul 27;14:1198585. doi: 10.3389/fphar.2023.1198585 (PMC10416097; doi:10.3389/fphar.2023.1198585)
Supplement: Supplementary file 1 [file DataSheet1.DOCX]

Supplementary Material

Cost-Effectiveness of Maintenance Niraparib with an Individualized Starting Dosage in Patients with Platinum-Sensitive Recurrent Ovarian Cancer in China

**Yin Shi†, Di Xiao†, Shuishi Li, Shao Liu*, Yu Zhang***

*** Correspondence:** Yu Zhang: [xyzhangyu@csu.edu.cn](mailto:xyzhangyu@csu.edu.cn), Or Shao Liu: liushao999@csu.edu.cn

**Supplementary Table S1.** Model parameters

**Supplementary Table S2.** Summary of statistical goodness-of-fit of Kaplan-Meier curve of niraparib and routine surveillance groups

**Supplementary Table S3.** Base-case result of scenario analyses (scenario 1)

**Supplementary Table S4.** Base-case result of scenario analyses (scenario 3)

**Supplementary Figure S1.** PFS Kaplan-Meier curve fitting and extrapolation

**Supplementary Figure S2.** OS Kaplan-Meier curve fitting and extrapolation

**Supplementary Figure S3.** Cost-effectiveness acceptability curves of scenario analysis (scenario 1) for the niraparib and routine surveillance groups in (A) the g*BRCA*m cohort and (B) the non-g*BRCA*m cohort generated from the probabilistic sensitivity analysis (10,000 iterations)

**Supplementary Figure S4.** Cost-effectiveness acceptability curves of scenario analysis (scenario 3) for the niraparib and routine surveillance groups in (A) the g*BRCA*m cohort and (B) the non-g*BRCA*m cohort generated from the probabilistic sensitivity analysis (10,000 iterations)

**Supplementary Table S1. Model parameters**

| **Parameter** | **Baseline Values** | **Range** | | **Distribution** | **Reference** |
| --- | --- | --- | --- | --- | --- |
|  |  | **lower values** | **upper values** |  |  |
| **Clinical inputs** | | | | | |
| Survival model for niraparib | | | | | |
| Log-normal model for PFS (g*BRCA*m) | μ=2.939566 σ=1.050669 |  |  |  | Model |
| Log-normal model for OS (g*BRCA*m) | μ=3.925271 σ=0.7020441 |  |  |  | Model |
| Log-normal model for PFS (non-g*BRCA*m) | μ=2.510452 σ=1.147878 |  |  |  | Model |
| Gamma model for OS (non-g*BRCA*m) | a=2.776989 b=0.05128088 |  |  |  | Model |
| Survival model for Placebo | | | | | |
| Log-logistic model for PFS (g*BRCA*m) | λ=0.01239383  γ=2.939343 |  |  |  | Model |
| Weibull model for OS (g*BRCA*m) | λ=0.0003363008 γ=1.956732 |  |  |  | Model |
| Log-normal model for PFS (non-g*BRCA*m) | μ=1.724008 σ=0.9255078 |  |  |  | Model |
| Gamma model for OS (non-g*BRCA*m) | a=2.037195 b=0.04040581 |  |  |  | Model |
| **Probability of grade ≥3 AEs** | | | | | |
| Neutropenia (Niraparib group) | 0.203 | 0.162 | 0.244 | Beta | [1] |
| Anemia (Niraparib group) | 0.147 | 0.118 | 0.176 | Beta | [1] |
| Thrombocytopenia (Niraparib group) | 0.113 | 0.090 | 0.136 | Beta | [1] |
| Neutropenia (Routine surveillance group) | 0.080 | 0.064 | 0.096 | Beta | [1] |
| Anemia (Routine surveillance group) | 0.023 | 0.018 | 0.028 | Beta | [1] |
| Thrombocytopenia (Routine surveillance group) | 0.011 | 0.009 | 0.013 | Beta | [1] |
| Proportion of patients receiving nilaparib at a dose of 200mg per day | 0.940 | 0.752 | 1.000 | Beta | [1] |
| Proportion of patients receiving nilaparib at a dose of 300mg per day | 0.060 | 0.048 | 0.072 | Beta | [1] |
| **Proportion of patients receiving subsequent treatment** | | | | | |
| Niraparib group | | | | | |
| Platinum plus bevacizumab | 0.046 | 0.037 | 0.055 | Beta | [2], clinical experience |
| Platinum | 0.408 | 0.327 | 0.490 | Beta | [2], clinical experience |
| Bevacizumab | 0.102 | 0.082 | 0.122 | Beta | [2], clinical experience |
| Paclitaxel albumin | 0.378 | 0.302 | 0.453 | Beta | [2], clinical experience |
| Niraparib | 0.051 | 0.041 | 0.061 | Beta | [2], clinical experience |
| Olaparib | 0.051 | 0.041 | 0.061 | Beta | [2], clinical experience |
| Letrozole | 0.046 | 0.037 | 0.055 | Beta | [2], clinical experience |
| Routine surveillance group | | | | | |
| Platinum plus bevacizumab | 0.040 | 0.032 | 0.048 | Beta | [2], clinical experience |
| Platinum | 0.394 | 0.315 | 0.473 | Beta | [2], clinical experience |
| Bevacizumab | 0.091 | 0.073 | 0.109 | Beta | [2], clinical experience |
| Paclitaxel albumin | 0.556 | 0.444 | 0.667 | Beta | [2], clinical experience |
| Niraparib (g*BRCA*m) | 0.270 | 0.216 | 0.324 | Beta | [2], clinical experience |
| Olaparib (g*BRCA*m) | 0.270 | 0.216 | 0.324 | Beta | [2], clinical experience |
| Niraparib (non-g*BRCA*) | 0.180 | 0.144 | 0.216 | Beta | [2], clinical experience |
| Olaparib (non-g*BRCA*) | 0.180 | 0.144 | 0.216 | Beta | [2], clinical experience |
| Letrozole | 0.061 | 0.048 | 0.073 | Beta | [2], clinical experience |
| **Rate of treatment discontinuation due to AEs** | | | | | |
| Niriparib group | 0.040 | 0.032 | 0.048 | Beta | [1] |
| Placebo group | 0.057 | 0.046 | 0.068 | Beta | [1] |
| **Cost inputs, $** | | | | | |
| Niraparib per mg | 0.24 | 0.05 | 0.24 | Gamma | [3], [4] |
| Paclitaxel per mg | 2.53 | 0.20 | 2.53 | Gamma | [3], [4] |
| Carboplatin per mg | 0.16 | 0.08 | 0.24 | Gamma | [3], [4] |
| Bevacizumab per mg | 2.33 | 1.56 | 2.33 | Gamma | [3], [4] |
| Doxorubicin liposome per mg | 35.51 | 30.64 | 36.11 | Gamma | [3], [4] |
| Paclitaxel albumin per mg | 1.16 | 1.07 | 1.21 | Gamma | [3], [4] |
| Olaparib per mg | 0.11 | 0.08 | 0.13 | Gamma | [3], [4] |
| Letrozole per mg | 0.22 | 0.14 | 1.58 | Gamma | [3], [4] |
| Managing neutropenia per event | 1280.62 | 1024.50 | 1536.74 | Gamma | Clinical experience |
| Managing anemia per event | 525.58 | 420.47 | 630.70 | Gamma | Clinical experience |
| Managing Thrombocytopenia per event | 1765.89 | 1412.71 | 2119.07 | Gamma | Clinical experience |
| Chemotherapy infusion | 72.35 | 57.88 | 86.82 | Gamma | Current local pricing |
| BRCA mutation testing | 465.12 | 372.09 | 558.14 | Gamma | Current local pricing |
| Terminal care | 1990.29 | 1592.23 | 2388.35 | Gamma | [5] |
| Follow up per cycle (PD) | 200.52 | 160.41 | 240.62 | Gamma | clinical experience |
| Follow up per cycle (PFS) | 109.51 | 87.61 | 131.41 | Gamma | clinical experience |
| **Utility inputs** | | | | | |
| PFS (Niraparib group) | 0.849 | 0.843 | 0.855 | Beta | [6] |
| PFS (Routine surveillance group) | 0.820 | 0.809 | 0.831 | Beta | [6] |
| PD (Niraparib group) | 0.793 | 0.772 | 0.813 | Beta | [6] |
| PD (Routine surveillance group) | 0.775 | 0.748 | 0.800 | Beta | [6] |
| **Other** | | | | | |
| Discount rate | 0.05 | 0.00 | 0.08 | Unifom | [7] |
| BSA | 1.64 | 1.312 | 1.968 | Normal | [1] |
| Body weight | 61.00 | 48.80 | 73.20 | Normal | [1] |

Abbreviation: PFS, progression-free survival; g*BRCA*m, germline *BRCA* mutation; OS, overall survival; AEs, adverse events; PD progressed disease; BSA, body surface area

**Supplementary Table S2. Summary of statistical goodness-of-fit of Kaplan-Meier curve of niraparib and** **routine surveillance groups**

|  | **Exponential** | **Weibull** | **Log-logistic** | **Lognormal** | **genGamma** | **Gamma** | **Gompertz** |
| --- | --- | --- | --- | --- | --- | --- | --- |
| **g*BRCA*m population** |  |  |  |  |  |  |  |
| PFS curve of niraparib group |  |  |  |  |  |  |  |
| AIC | 227.8415 | 226.7371 | 225.0066 | 223.4579 | 224.5838 | 225.8974 | 229.2941 |
| BIC | 230.0159 | 231.0858 | 229.3554 | 227.8066 | 231.1070 | 230.2462 | 233.6429 |
| PFS curve of routine surveillance group |  |  |  |  |  |  |  |
| AIC | 168.5126 | 162.4224 | 153.5248 | 154.2337 | 155.5848 | 158.1595 | 169.5412 |
| BIC | 169.8799 | 165.1570 | 156.2594 | 156.9683 | 159.6867 | 160.8941 | 172.2758 |
| OS curve of niraparib group |  |  |  |  |  |  |  |
| AIC | 257.8866 | 247.0179 | 244.6295 | 243.0431 | 243.8036 | 245.0644 | 252.7493 |
| BIC | 260.0763 | 251.3972 | 249.0088 | 247.4224 | 250.3726 | 249.4437 | 257.1287 |
| OS curve of routine surveillance group |  |  |  |  |  |  |  |
| AIC | 154.0255 | 149.3144 | 149.6221 | 150.1611 | 151.2683 | 149.4835 | 149.9151 |
| BIC | 155.6890 | 152.6415 | 152.9492 | 153.4882 | 156.2590 | 152.8106 | 153.2422 |
| **non-g*BRCA*m population** |  |  |  |  |  |  |  |
| PFS curve of niraparib group |  |  |  |  |  |  |  |
| AIC | 517.0122 | 517.6900 | 510.7494 | 508.2310 | 507.9505 | 516.4271 | 518.7719 |
| BIC | 519.7658 | 523.1972 | 516.2566 | 513.7382 | 516.2112 | 521.9342 | 524.2791 |
| PFS curve of routine surveillance group |  |  |  |  |  |  |  |
| AIC | 270.4137 | 271.7223 | 259.1345 | 258.4911 | 243.8482 | 269.9057 | 271.3187 |
| BIC | 272.3649 | 275.6248 | 263.0370 | 262.3935 | 249.7020 | 273.8082 | 275.2212 |
| OS curve of niraparib group |  |  |  |  |  |  |  |
| AIC | 454.0843 | 434.5758 | 433.4065 | 431.6158 | 433.3242 | 433.1062 | 440.5710 |
| BIC | 456.7756 | 439.9585 | 438.7891 | 436.9985 | 441.3982 | 438.4889 | 445.9537 |
| OS curve of routine surveillance group |  |  |  |  |  |  |  |
| AIC | 232.6227 | 228.8486 | 227.6209 | 226.2748 | 227.4894 | 227.9748 | 231.7758 |
| BIC | 234.5740 | 232.7511 | 231.5233 | 230.1773 | 233.3431 | 231.8773 | 235.6783 |

Abbreviations: g*BRCA*m, germline *BRCA* mutation; OS, overall survival; PFS, progression-free survival; AIC, Akaike’s information criterion; BIC, Bayesian information criterion.

The visual fits and statistical fits of seven parametric survival models including exponential, Weibull, log-logistic, lognormal, genGamma, Gamma, and Gompertz are presented in eTable 2, eFigure 1, and eFigure 2.

In the g*BRCA*m cohort, the best-fit distribution for the niraparib PFS curve was chosen according to the lowest value of the AIC and BIC. For the niraparib PFS curve, routine surveillance PFS curve, niraparib OS curve, and routine surveillance OS curve, the best-fit distribution was lognormal, log-logistic, lognormal, and Weibull, respectively.

In the non-g*BRCA*m cohort, As for the niraparib PFS curve, genGamma and lognormal distribution had the lowest AIC and BIC, respectively. However, BIC is more credible with a bigger penalty, and AIC is preferred for large samples. Above, the genGamma distribution was likely to be the most reasonable parametric model for the PFS of niraparib. As for the routine surveillance PFS curve, the genGamma distribution had the lowest AIC and BIC for PFS of routine surveillance. Therefore, genGamma distribution may be appropriate for PFS of routine surveillance. As for the niraparib and routine surveillance OS curves, lognormal had the lowest AIC and BIC. However, the lognormal models typically have long tails due to a reducing hazard as time increases after a certain point, which would likely overestimate OS in the long term based on clinical experts’ opinions. Therefore, gamma distribution may be more appropriate for the OS curve of niraparib and routine surveillance.

**Supplementary Table S3. Base-case result of scenario analyses (scenario 1)**

| **Strategies** | **Cost, $** | **QALYs** | **LYs** | **ICER, $/QALY** | **ICER, $/LY** |
| --- | --- | --- | --- | --- | --- |
| **gBRCAm** |  |  |  |  |  |
| Niraparib | 43,933.14 | 3.39 | 4.15 | 33,009.06 | 34,319.59 |
| Placebo | 24,714.17 | 2.80 | 3.59 |  |  |
| **Non-gBRCAm** |  |  |  |  |  |
| Niraparib | 37,205.66 | 2.96 | 3.65 | 62,763.81 | 74,479.89 |
| Placebo | 17,840.89 | 2.66 | 3.39 |  |  |

Abbreviations: QALY, quality-adjusted life-years; gBRCAm, germline BRCA mutation; ICER, Incremental cost-effectiveness ratio.

**Supplementary Table S4. Base-case result of scenario analyses (scenario 3)**

| **Strategies** | **Cost, $** | **QALYs** | **LYs** | **ICER, $/QALY** | **ICER, $/LY** |
| --- | --- | --- | --- | --- | --- |
| **gBRCAm** |  |  |  |  |  |
| Niraparib | 14,868.68 | 3.39 | 4.15 | 3,372.19 | 3,506.07 |
| Placebo | 12905.28 | 2.80 | 3.59 |  |  |
| **Non-gBRCAm** |  |  |  |  |  |
| Niraparib | 13,309.74 | 2.96 | 3.65 | 5,643.96 | 6,697.50 |
| Placebo | 11,568.39 | 2.66 | 3.39 |  |  |

Abbreviations: QALY, quality-adjusted life-years; gBRCAm, germline BRCA mutation; ICER, Incremental cost-effectiveness ratio.

**Supplementary Figure S1.** **PFS** **Kaplan-Meier curve fitting and extrapolation.**


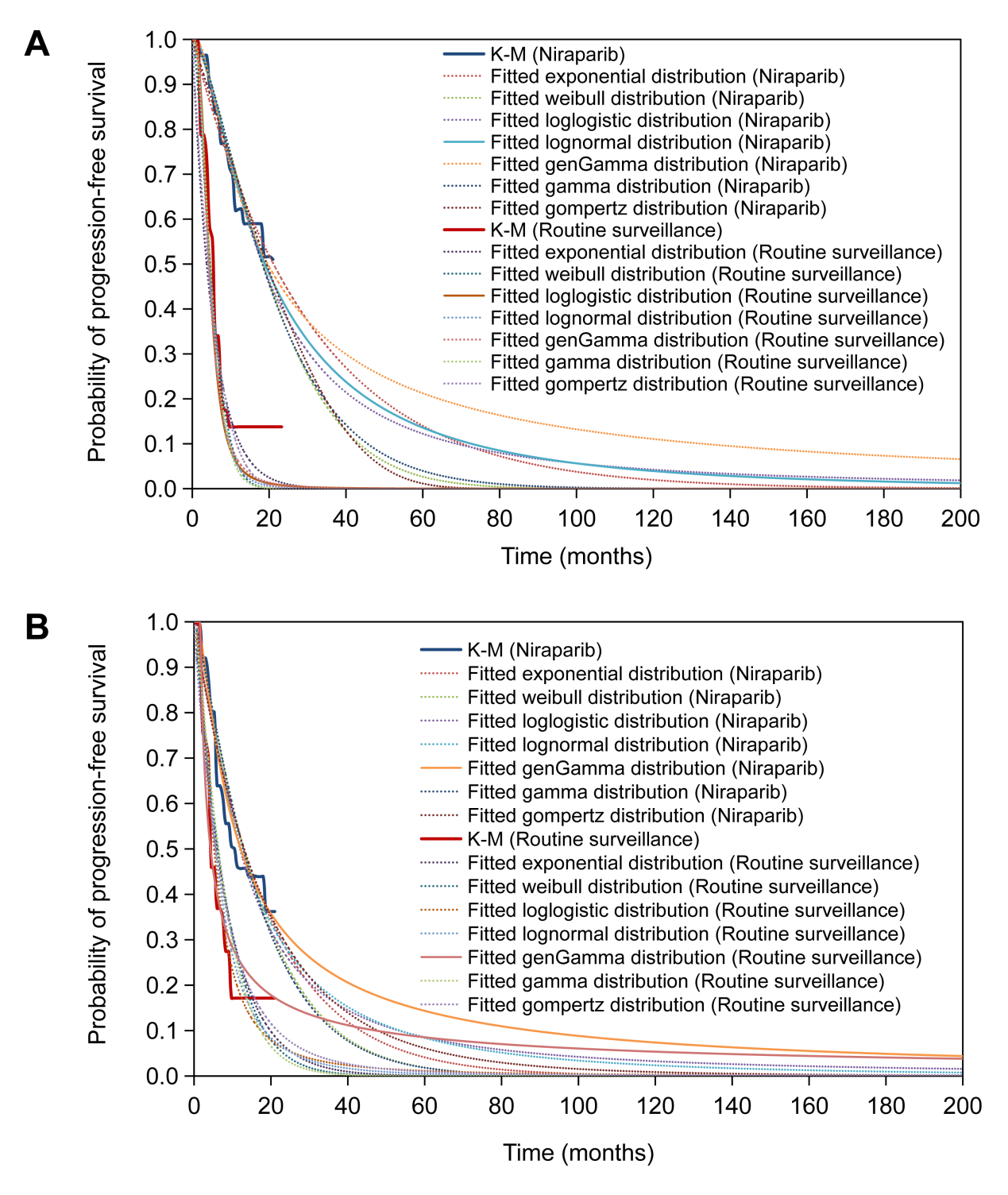


Abbreviations: PFS, progression-free survival; K-M, Kaplan-Meier

**Supplementary Figure S2.** **OS Kaplan-Meier curve fitting and extrapolation.**


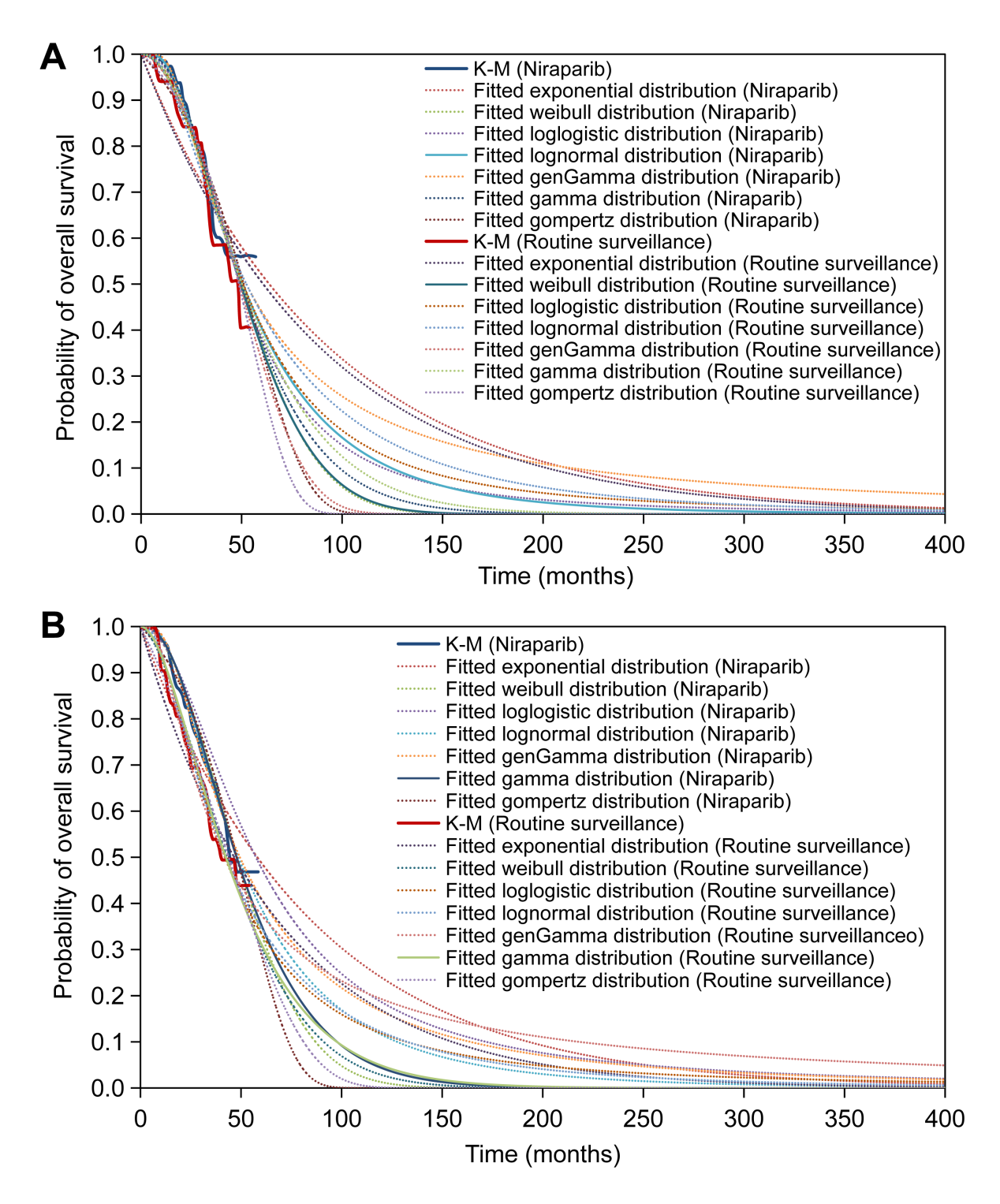


Abbreviations: OS, overall survival; K-M, Kaplan-Meier

**Supplementary Figure S3.** **Cost-effectiveness acceptability curves of scenario analysis (scenario 1) for the niraparib and routine surveillance groups in (A) the gBRCAm cohort and (B) non-gBRCAm cohort generated from the probabilistic sensitivity analysis (10,000 iterations).**


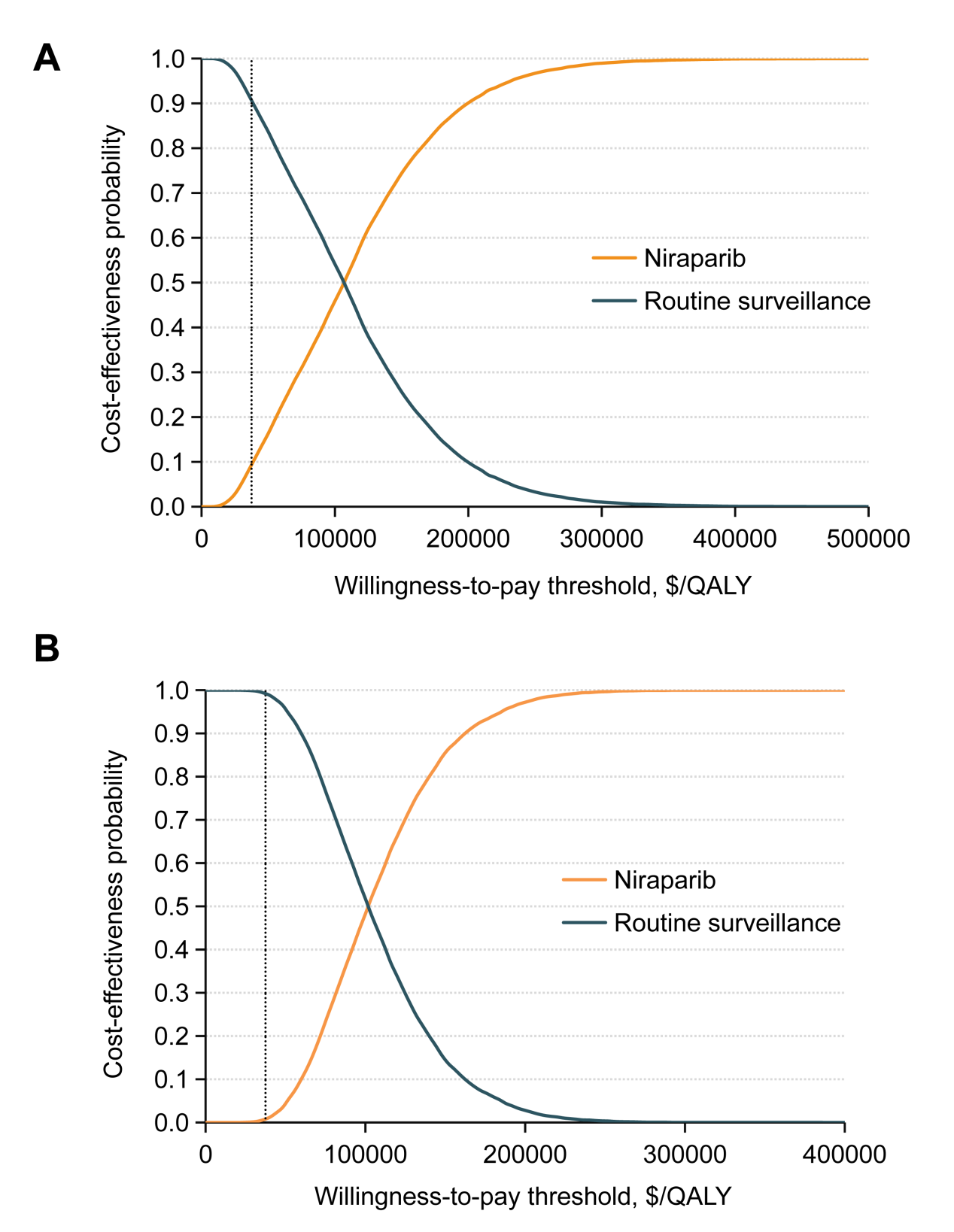


The black vertical dotted line represents the $37,488 per QALY. Abbreviations: QALY, quality-adjusted life-years; gBRCAm, germline BRCA mutation

**Supplementary Figure S4.** **Cost-effectiveness acceptability curves of scenario analysis (scenario 3) for the niraparib and routine surveillance groups in (A) the gBRCAm cohort and (B) non-gBRCAm cohort generated from the probabilistic sensitivity analysis (10,000 iterations).**


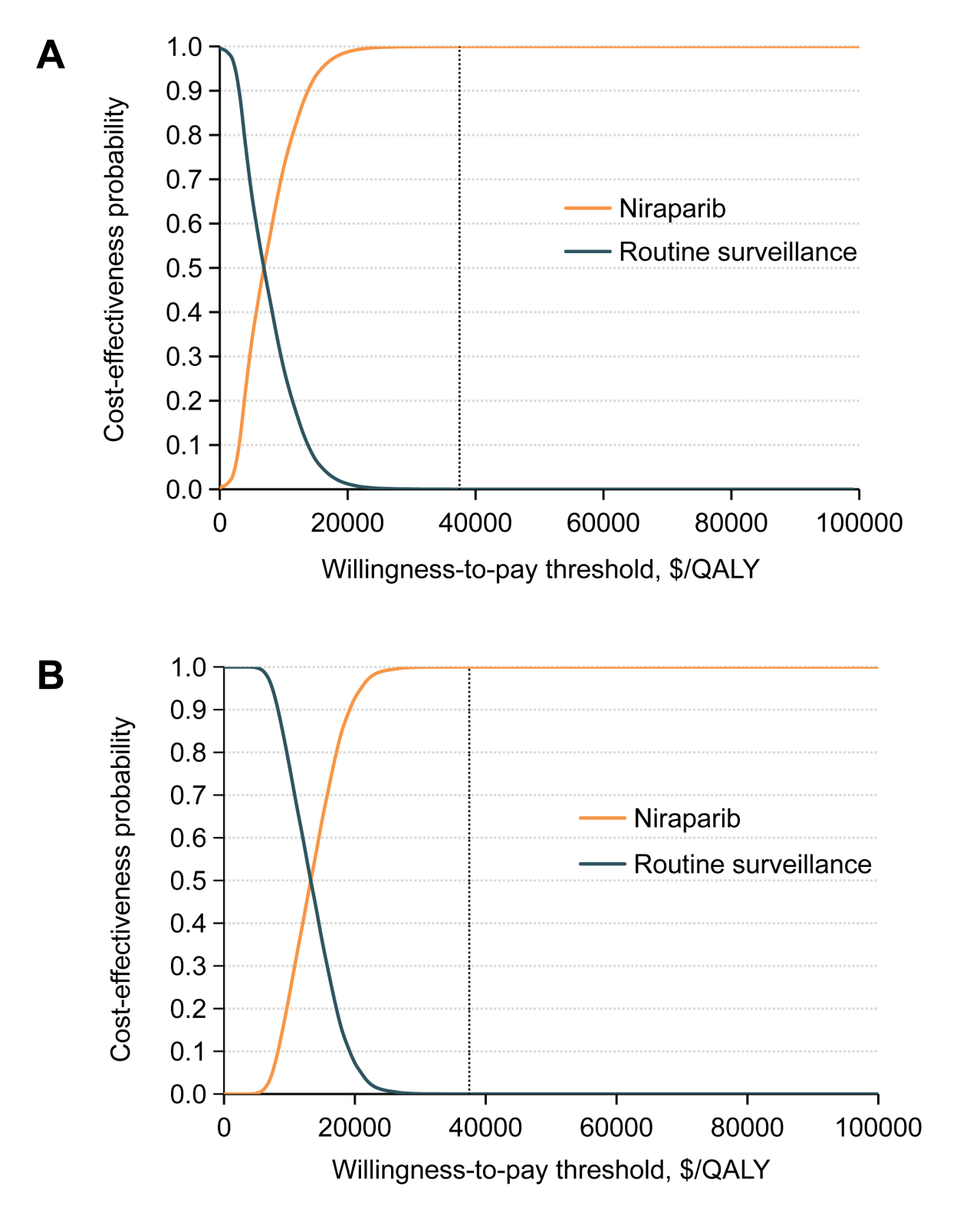


The black vertical dotted line represents the $37,488 per QALY. Abbreviations: QALY, quality-adjusted life-years; gBRCAm, germline BRCA mutation

**References**

[1] Wu XH, Zhu JQ, Yin RT, Yang JX, Liu JH, Wang J, et al. Niraparib maintenance therapy in patients with platinum-sensitive recurrent ovarian cancer using an individualized starting dose (NORA): a randomized, double-blind, placebo-controlled phase III trial. Ann Oncol. 2021;32:512-21.

[2] Poveda A, Floquet A, Ledermann JA, Asher R, Penson RT, Oza AM, et al. Olaparib tablets as maintenance therapy in patients with platinum-sensitive relapsed ovarian cancer and a BRCA1/2 mutation (SOLO2/ENGOT-Ov21): a final analysis of a double-blind, randomised, placebo-controlled, phase 3 trial. Lancet Oncol. 2021;22:620-31.

[3] The State Council the People's Republic of China. National Drug Catalogue for Basic Medical Insurance, Work-related Injury Insurance and Maternity Insurance. <http://www.gov.cn/zhengce/zhengceku/2021-12/03/content_5655651.htm>, 2021 (Accessed July 15 2022).

[4] Hunan Public Resources Trading Center. Pharmaceutical Classified Procurement System of Hunan Province. https://jyjy.hnsggzy.com/, 2022 (Accessed July 15 2022).

[5] Li H, Lai L, Wu B. Cost Effectiveness of Ceritinib and Alectinib Versus Crizotinib in First-Line Anaplastic Lymphoma Kinase-Positive Advanced Non-small-cell Lung Cancer. Clinical drug investigation. 2020;40:183-9.

[6] Guy H, Walder L, Fisher M. Cost-Effectiveness of Niraparib Versus Routine Surveillance, Olaparib and Rucaparib for the Maintenance Treatment of Patients with Ovarian Cancer in the United States. Pharmacoeconomics. 2019;37:391-405.

[7] Chinese Pharmaceutical Association. China Guidelines for Pharmcoeconomic Evaluation(2020). https://www.ispor.org/heor-resources/more-heor-resources/pharmacoeconomic-quidelines/pe-quideline-detail/china-mainland, 2021 (Accessed Apr 22 2021).
